# Erratum: Comparison of R9.4.1/Kit10 and R10/Kit12 Oxford Nanopore flowcells and chemistries in bacterial genome reconstruction

**DOI:** 10.1099/mgen.0.001144

**Published:** 2023-11-28

**Authors:** Nicholas D. Sanderson, Natalia Kapel, Gillian Rodger, Hermione Webster, Samuel Lipworth, Teresa L. Street, Timothy Peto, Derrick Crook, Nicole Stoesser

**Affiliations:** ^1^​ NIHR OxfordBiomedical Research Centre, University of Oxford, Oxford, UK; ^2^​ Nuffield Department of Medicine, University of Oxford, Oxford, UK; ^3^​ NIHR Health Protection Research Unit in Healthcare Associated Infections and Antimicrobial Resistance at University of Oxford in partnership with Public Health England, Oxford, UK

In the published version of the article, the shading in Table 2 containing information described in the legends and text has not carried over into the final version. The correct table can be found below:

**Table 2**. Number of unique contigs by sequencing modality and bacterial species using the complete data available (i.e. no subsampling). The first number in each cell represents the number of contigs assembled and matching to the Illumina-corrected reference using dnadiff, the total number of contigs assembled is shown in curved brackets, and the proportion of the reference chromosomal contig covered in square brackets. For *E. coli*, *P. aeruginosa*, and *S. aureus* the total number of expected contigs is 1, for *K. pneumoniae* the expected number of contigs is 1 chromosome + 5 plasmids. Orange shading shows absent contigs, and/or incomplete assembly (n >1 contig matching to reference), and/or extra contigs not matching to reference. Green shaded cells denote complete singular contigs which reflect the reference DNA content at 100+/-1%. “-“ denotes no relevant contig assembled.

**Table:** 

Sequencing modality	Assembler	Plexed Y/N	*E. coli*, chromosome (1) 5 231 428 bp 51 % GC	*P.* *aeruginosa*, chromosome (1) 6 264 404 bp 66.2 %GC	*S.* *aureus*, chromosome (1) 2 902 619 bp 32.8 %GC	*K.* *pneumoniae*, chromosome (1) 5 315 120 bp 57.0 %GC	*K.* *pneumoniae*, pKPN3 plasmid (1) 175 879 51.7 %GC	*K.* *pneumoniae*, pKPN4 plasmid (1) 107576 bp 53.4 %GC	*K.* *pneumoniae*, pKPN5 (plasmid (1) 88 582 bp 53.8 %GC	*K.* *pneumoniae*, pKPN6 (plasmid (1) 4259 bp 41.4 %GC	*K.* *pneumoniae*, pKPN7 (plasmid (1) 3478 bp 45.7 %GC
Illumina	SPAdes	Y	226 (326) [98.64 %]	115 (152) [99.72 %]	86 (150) [98.42 %]	117 (312) [98.9 %]	41 [79.7 %]	45 [100.72 %]	22 [82.49 %]	1 [102.82 %]	1 [103.45 %]
R9.4.1 hac +Illumina	Unicycler	Y	1 (1) [100.09 %]	1 (1) [100.49 %]	1 (1) [100.69 %]	1 (7) [100.1 %]	1 [100 %]	1 [100 %]	1 [100 %]	2 [100 %]	1 [96.15 %]
R9.4.1 hac	Canu	Y	1 (1) [100.59 %]	1 (1) [101.29 %]	1 (1) [103.18 %]	1 (24) [100.72 %]	1 [131.55 %]	1 [172.51 %]	1 [100 %]	2 [133.5 %]	1 [100 %]
R9.4.1 sup	Canu	Y	1 (1) [100.65 %]	1 (1) [101.18 %]	1 (1) [102.43 %]	1 (7) [100.96 %]	1 [129.41 %]	1 [100 %]	1 [125.07 %]	1 [100 %]	1 [100 %]
R10.3 hac	Canu	N	1 (6) [100.49 %]	1 (2) [101.19 %]	1 (3) [102.78 %]	1 (8) [100.72 %]	1 [100 %]	1 [100 %]	1 [100 %]	1 [100 %]	1 [100 %]
R10.3 sup	Canu	N	1 (2) [100.6 %]	1 (3) [101.13 %]	1 (3) [102.85]	1 (8) [100.74 %]	1 [100 %]	1 [138.13 %]	1 [143.94 %]	1 [192.58 %]	1 [100 %]
R10.4 hac	Canu	N	1 (6) [100.62 %]	1 (1) [101.06 %]	1 (2) [103.6 %]	1 (8) [100.8 %]	1 [127.89 %]	1 [141.81 %]	1 [147.6 %]	1 [100 %]	1 [112.85 %]
R10.4 sup	Canu	N	1 (3) [100.61 %]	1 (2) [100.1 %]	1 (2) [102.33 %]	1 (7) [100 %]	1 [100.94 %]	1 [100 %]	1 [145.43 %]	1 [100 %]	1 [100 %]
R10.4 sup duplex	Canu	N	4 (42) [100.12 %]	1 (14) [101.5 %]	1 (29) [101.72 %]	1 (41) [100.1 %]	1 [100 %]	3 [130.98 %]	1 [149.19 %]	1 [100 %]	1 [100 %]
R10.4 hac	Canu	Y	1 (2) [100.42 %]	1 (1) [100.87 %]	1 (1) [102.01 %]	1 (7) [100.82 %]	1 [124.01 %]	1 [100 %]	1 [142.89 %]	1 [100 %]	1 [100 %]
R10.4 sup	Canu	Y	1 (1) [100.48 %]	1 (1) [100.17 %]	1 (1) [102.29 %]	1 (12) [100.61 %]	1 [117.3 %]	2 [134.06 %]	1 [137.68 %]	1 [100 %]	1 [100 %]
R10.4 sup duplex	Canu	Y	-*	23 (25) [99.2 %]	-*	15 (25) [99.2 %]	1 [80.39 %]	2 [97.73 %]	1 [112.86 %]	1 [94.01 %]	1 [100 %]
R9.4.1 hac	Flye	Y	1 (1) [100.09 %]	1 (1) [100.14 %]	1 (1) [100.7 %]	1 (4) [100.11 %]	1 [75.7 %]	1 [103.68 %]	1 [100 %]	–	–
R9.4.1 sup	Flye	Y	1 (1) [100.09 %]	1 (1) [100.47 %]	1 (1) [100 %]	1 (5) [100.1 %]	1 [100 %]	1 [99.99 %]	1 [100 %]	–	–
R10.3 hac	Flye	N	1 (1) [100.10 %]	1 (1) [100.44 %]	1 (1) [100.69 %]	1 (4) [100.1 %]	1 [95.83 %]	1 [73.13 %]	1 [100 %]	–	–
R10.3 sup	Flye	N	1 (1) [100.09 %]	1 (1) [100.44 %]	1 (1) [100.69 %]	1 (4) [100.1 %]	1 [100 %]	1 [100 %]	1 [100 %]	–	–
R10.4 hac	Flye	N	1 (1) [100.10 %]	1 (1) [100.11 %]	1 (1) [100.69 %]	1 (4) [100.1 %]	1 [100 %]	1 [100 %]	1 [100 %]	–	–
R10.4 sup	Flye	N	1 (1) [100.09 %]	1 (1) [100.4 %]	1 (1) [100.69%]	1 (5) [100.1 %]	1 [100 %]	1 [98.92 %]	1 [99.99 %]	–	–
R10.4 sup duplex	Flye	N	1 (3) [100.10 %]	1 (1) [100.8 %]	1 (2) [100.69 %]	1 (7) [100.21 %]	1 [94.27 %]	1 [102.93 %]	1 [101.54 %]	–	1 [100 %]
R10.4 hac	Flye	Y	1 (1) [100.10 %]	1 (1) [100.16 %]	1 (1) [100.69 %]	1 (5) [100.1 %]	1 [100 %]	1 [100 %]	1 [100 %]	–	–
R10.4 sup	Flye	Y	1 (1) [100.00 %]	1 (1) [100.48 %]	1 (1) [100.69 %]	1 (5) [100.11 %]	1 [100 %]	1 [100 %]	1 [100 %]	–	–
R10.4 sup duplex	Flye	Y	37 (38) [8.47 %]	1 (5) [100.48 %]	25 (25) [83.64 %]	1 (4) [100.4 %]	1 [84.71 %]	1 [105.27 %]	1 [100 %]	–	–

*Insufficient read depth for canu to assemble using default settings.

The Microbiology Society apologises for any inconvenience caused.

