# Supplementary material for: Comparison of R9.4.1/Kit10 and R10/Kit12 Oxford Nanopore flowcells and chemistries in bacterial genome reconstruction
Source: Microb Genom. 2023 Jan 10;9(1):mgen000910. doi: 10.1099/mgen.0.000910 (PMC9973852; doi:10.1099/mgen.0.000910)

Supplementary material

Table S1. DNA concentrations at initial extraction. Measurements taken on 13/Sep/2021.

**Well**	**DIN**	**Concentration (ng/microL)**	**Sample description**	**Alert**	**Observations**	**Notes**
A1	-	80.0	Ladder		Ladder	
B1	9.1	438	*Pseudomonas aeruginosa* PA01	!	Sample concentration outside functional range for DIN and the assay	
C1	8.9	198	*Klebsiella pneumoniae* MGH78578	!	Sample concentration outside functional range for DIN and the assay	
D1	9.3	264	Escherichia coli CFT073	!	Sample concentration outside functional range for DIN and the assay	
E1	9.2	314	*Staphylococcus aureus* MRSA252 - extract 1	!	Sample concentration outside functional range for DIN and the assay	Not used
F1	9.1	295	*Staphylococcus aureus* MRSA252 - extract 2	!	Sample concentration outside functional range for DIN and the assay	MRSA2 was chosen for all further experiments (extracted twice due to problems with lysis)

Table S2. DNA concentrations at initial extraction, diluted 10x. Measurements taken on 13/Sep/2021.

**Well**	**DIN**	**Concentration (ng/microL)**	**Sample description**	**Alert**	**Observations**
A1	-	73.1	Ladder		Ladder
B1	9.1	22.8	*Pseudomonas aeruginosa* PA01		
C1	8.9	23.7	*Klebsiella pneumoniae* MGH78578		
D1	9.3	25.0	Escherichia coli CFT073		
E1	9.2	25.8	*Staphylococcus aureus* MRSA252 - extract 1		Not used
F1	9.1	36.4	*Staphylococcus aureus* MRSA252 - extract 2		

Table S3. DNA concentrations after final experiments, taken on 31/Jan/2022. To verify quality after long-term storage at 4°C.

Well	DIN	Concentration (ng/microL)	Sample description	Alert	Observations	Notes
A1	-	51.9	Ladder		Ladder	
B1	9.4	28.3	*Pseudomonas aeruginosa* PA01 - diluted 10x			
C1	9.4	20.8	*Klebsiella pneumoniae* MGH78578 - diluted 10x			
D1	9.4	34.1	*Escherichia coli* CFT073 - diluted 10x			
E1	9.7	48.9	*Staphylococcus aureus* MRSA252 - extract 2 - diluted 10x			
F1	9.1	210	*Pseudomonas aeruginosa* Pa01 - neat DNA	!	Sample concentration outside functional range for DIN and the assay	
G1	9.2	220	*Klebsiella pneumoniae* MGH78578 - neat DNA	!	Sample concentration outside functional range for DIN and the assay	
H1	9.2	307	*Escherichia coli* CFT073	!	Sample concentration outside functional range for DIN and the assay	
A2	8.7	426	*Staphylococcus aureus* MRSA252 - extract 2 - neat DNA	!	Sample concentration outside functional range for DIN and the assay	
B2	9.1	238	*Staphylococcus aureus* MRSA252 - extract 1 - neat DNA	!	Sample concentration outside functional range for DIN and the assay	Not used

Table S4. Qubit DNA concentrations over time.

		Qubit concentration (microg/mL)			
**Date**	**Measurement**	***E. coli***	***K. pneumoniae***	***P. aeruginosa***	***S. aureus* (extract 2)**
02/Sep/2021	Post-extraction	586	364	320	NA
03/Sep/2021	Post-extraction	NA	NA	NA	302
21/Sep/2021	R10.3 sequencing	330	300	456	374
23/Nov/2021	R10.4 sequencing	266	246	NA	296
25/Nov/2021	R10.4 sequencing	NA	NA	356	NA
24/Jan/2021	Concentration check	386	289	402	322
11/Feb/2022	R10.4 multiplex sequencing	274	248	334	312

Table S5. Flowcell use and pore count QC for each flowcell

Flow Cell number	Flow Cell/Chemistry	Pore count pre-sequencing	Organism
FAR12872	9.4/Kit10	1187	*E. coli + K. pneumoniae + P. aeruginosa + S. aureus* - multiplexed (rapid barcoding)
FAQ96597	10.3/Kit12	1296	*P. aeruginosa*
FAQ91874	10.3/Kit12	1374	*K. pneumoniae*
FAQ91081	10.3/Kit12	1250	*E. coli*
FAQ96358	10.3/Kit12	1242	*S. aureus*
FAR04200	10.4/Kit12	1434	*E. coli*
FAR28144	10.4/Kit12	1526	*K. pneumoniae*
FAR28230	10.4/Kit12	1598	*S. aureus*
FAR28230 - after wash	10.4/Kit12	1126	*P. aeruginosa*
FAS62700	10.4/Kit12	1275	*E. coli + K. pneumoniae + P. aeruginosa + S. aureus* - multiplexed (native barcoding)

Table S6. Median and modal read accuracies for all modality, flowcell/kit and basecalling combinations. Accuracies are evaluated against the relevant Illumina-corrected reference.

Sequence type	Plexed (yes/ no)	Flowcell/ chemistry	Species	Read type/basecaller	Median % accuracy	Modal % accuracy
Illumina	Yes	NA	*E. coli*	NA	100.0	100.0
Nanopore	Yes	R9.4.1	*E. coli*	hac	96.2	97.2
Nanopore	Yes	R9.4.1	*E. coli*	sup	95.8	96.7
Nanopore	No	R10.3	*E. coli*	hac	96.9	98.1
Nanopore	No	R10.3	*E. coli*	sup	96.4	97.8
Nanopore	No	R10.4	*E. coli*	hac	95.7	96.5
Nanopore	No	R10.4	*E. coli*	sup	97.5	98.3
Nanopore	No	R10.4	*E. coli*	duplex/sup	99.8	99.9
Nanopore	Yes	R10.4	*E. coli*	hac	97.1	97.8
Nanopore	Yes	R10.4	*E. coli*	sup	98.5	99.0
Nanopore	Yes	R10.4	*E. coli*	duplex/sup	99.9	100.0
Illumina	Yes	NA	*K. pneumoniae*	NA	100.0	100.0
Nanopore	Yes	R9.4.1	*K. pneumoniae*	hac	96.2	97.0
Nanopore	Yes	R9.4.1	*K. pneumoniae*	sup	95.7	96.7
Nanopore	No	R10.3	*K. pneumoniae*	hac	97.4	98.2
Nanopore	No	R10.3	*K. pneumoniae*	sup	97.0	97.9
Nanopore	No	R10.4	*K. pneumoniae*	hac	96.1	97.1
Nanopore	No	R10.4	*K. pneumoniae*	sup	97.9	98.5
Nanopore	No	R10.4	*K. pneumoniae*	duplex/sup	99.9	99.9
Nanopore	Yes	R10.4	*K. pneumoniae*	hac	97.3	97.9
Nanopore	Yes	R10.4	*K. pneumoniae*	sup	98.6	99.2
Nanopore	Yes	R10.4	*K. pneumoniae*	duplex/sup	99.9	99.9
Illumina	Yes	NA	*P. aeruginosa*	NA	100.0	100.0
Nanopore	Yes	R9.4.1	*P. aeruginosa*	hac	96.6	97.2
Nanopore	Yes	R9.4.1	*P. aeruginosa*	sup	96.5	97.2
Nanopore	No	R10.3	*P. aeruginosa*	hac	98.3	98.9
Nanopore	No	R10.3	*P. aeruginosa*	sup	97.9	98.5
Nanopore	No	R10.4	*P. aeruginosa*	hac	96.6	97.4
Nanopore	No	R10.4	*P. aeruginosa*	sup	98.4	98.9
Nanopore	No	R10.4	*P. aeruginosa*	duplex/sup	99.8	99.9
Nanopore	Yes	R10.4	*P. aeruginosa*	hac	97.7	98.2
Nanopore	Yes	R10.4	*P. aeruginosa*	sup	99.0	99.4
Nanopore	Yes	R10.4	*P. aeruginosa*	duplex/sup	99.9	100.0
Illumina	Yes	NA	*S. aureus*	NA	100.0	100.0
Nanopore	Yes	R9.4.1	*S. aureus*	hac	97.0	97.8
Nanopore	Yes	R9.4.1	*S. aureus*	sup	96.8	97.4
Nanopore	No	R10.3	*S. aureus*	hac	98.3	99.0
Nanopore	No	R10.3	*S. aureus*	sup	98.1	98.7
Nanopore	No	R10.4	*S. aureus*	hac	97.3	98.0
Nanopore	No	R10.4	*S. aureus*	sup	98.7	99.1
Nanopore	No	R10.4	*S. aureus*	duplex/sup	99.9	100.0

Nanopore	Yes	R10.4	*S. aureus*	hac	98.3	99.0
Nanopore	Yes	R10.4	*S. aureus*	sup	99.4	99.9
Nanopore	Yes	R10.4	*S. aureus*	duplex/sup	100.0	100.0

Table S7. Number of SNPs and indels observed by sequencing data type, basecalling and assembler type. Values representing ≤1 error/100kb of sequence are shaded in green. Numbers shown are using all read data evaluated for each modality, and for unplexed R10.4.1 runs. An average reference genome size of 4,928,392bp was used to estimate the median number of errors per 100kb of sequence.

**Sequence type**	**Plexed (yes/ no)**	**Flowcell/ chemistry**	**Read type/basecaller**	**Assembler**	**Error type (SNPs/indels)**	**Median number of error/100kb of sequence (SNPs or indels)**
SNPs						
Illumina	Yes	NA	NA	SPAdes	SNPs	0.71
Nanopore	Yes	R9.4.1	hac	Canu	SNPs	1.03
Nanopore	Yes	R9.4.1	hac	Flye_hq	SNPs	1.72
Nanopore	Yes	R9.4.1	hac	Flye_hq+ medaka 1x	SNPs	0.59
Nanopore	Yes	R9.4.1	hac	Flye_hq+ medaka 2x	SNPs	0.57
Nanopore	Yes	R9.4.1	hac	Flye_hq+ medaka 3x	SNPs	0.56
Nanopore	Yes	R9.4.1	sup	Canu	SNPs	1.24
Nanopore	Yes	R9.4.1	sup	Flye_hq	SNPs	2.08
Nanopore	Yes	R9.4.1	sup	Flye_hq+ medaka 1x	SNPs	0.67
Nanopore	Yes	R9.4.1	sup	Flye_hq+ medaka 2x	SNPs	0.69
Nanopore	Yes	R9.4.1	sup	Flye_hq+ medaka 3x	SNPs	0.68
Nanopore+ Illumina	Yes	R9.4.1	hac	Unicycler	SNPs	4.38
Nanopore+ Illumina	Yes	R9.4.1	sup	Unicycler	SNPs	4.38
Nanopore	No	R10.3	hac	Canu	SNPs	0.78
Nanopore	No	R10.3	hac	Flye_hq	SNPs	1.74
Nanopore	No	R10.3	hac	Flye_hq+ medaka 1x	SNPs	0.23
Nanopore	No	R10.3	hac	Flye_hq+ medaka 2x	SNPs	0.21
Nanopore	No	R10.3	hac	Flye_hq+ medaka 3x	SNPs	0.21
Nanopore	No	R10.3	sup	Canu	SNPs	0.89
Nanopore	No	R10.3	sup	Flye_hq	SNPs	1.73
Nanopore	No	R10.3	sup	Flye_hq+ medaka 1x	SNPs	0.92
Nanopore	No	R10.3	sup	Flye_hq+	SNPs	0.93

				medaka 2x		
Nanopore	No	R10.3	sup	Flye_hq+ medaka 3x	SNPs	0.93
Nanopore	No	R10.4	hac	Canu	SNPs	1.71
Nanopore	No	R10.4	hac	Flye_hq	SNPs	3.12
Nanopore	No	R10.4	hac	Flye_hq+ medaka 1x	SNPs	0.71
Nanopore	No	R10.4	hac	Flye_hq+ medaka 2x	SNPs	0.73
Nanopore	No	R10.4	hac	Flye_hq+ medaka 3x	SNPs	0.69
Nanopore	No	R10.4	sup	Canu	SNPs	1.23
Nanopore	No	R10.4	sup	Flye_hq	SNPs	2.77
Nanopore	No	R10.4	sup	Flye_hq+ medaka 1x	SNPs	1.02
Nanopore	No	R10.4	sup	Flye_hq+ medaka 2x	SNPs	1.06
Nanopore	No	R10.4	sup	Flye_hq+ medaka 3x	SNPs	1.02
Nanopore	No	R10.4	duplex/sup	Canu	SNPs	0.26
Nanopore	No	R10.4	duplex/sup	Flye_hq	SNPs	0.27
Nanopore	No	R10.4	duplex/sup	Flye_hq+ medaka 1x	SNPs	0.21
Nanopore	No	R10.4	duplex/sup	Flye_hq+ medaka 2x	SNPs	0.21
Nanopore	No	R10.4	duplex/sup	Flye_hq+ medaka 3x	SNPs	0.21
Indels						
Illumina	Yes	NA	NA	SPAdes	Indels	0.02
Nanopore	Yes	R9.4.1	hac	Canu	Indels	26.83
Nanopore	Yes	R9.4.1	hac	Flye_hq	Indels	17.57
Nanopore	Yes	R9.4.1	hac	Flye_hq+ medaka 1x	Indels	2.97
Nanopore	Yes	R9.4.1	hac	Flye_hq+ medaka 2x	Indels	3.37
Nanopore	Yes	R9.4.1	hac	Flye_hq+ medaka 3x	Indels	3.00
Nanopore	Yes	R9.4.1	sup	Canu	Indels	25.83
Nanopore	Yes	R9.4.1	sup	Flye_hq	Indels	17.56
Nanopore	Yes	R9.4.1	sup	Flye_hq+ medaka 1x	Indels	3.17
Nanopore	Yes	R9.4.1	sup	Flye_hq+ medaka	Indels	3.23

				2x		
Nanopore	Yes	R9.4.1	sup	Flye_hq+ medaka 3x	Indels	3.13
Nanopore+ Illumina	Yes	R9.4.1	hac	Unicycler	Indels	0.56
Nanopore+ Illumina	Yes	R9.4.1	sup	Unicycler	Indels	0.57
Nanopore	No	R10.3	hac	Canu	Indels	3.22
Nanopore	No	R10.3	hac	Flye_hq	Indels	1.79
Nanopore	No	R10.3	hac	Flye_hq+ medaka 1x	Indels	0.92
Nanopore	No	R10.3	hac	Flye_hq+ medaka 2x	Indels	0.50
Nanopore	No	R10.3	hac	Flye_hq+ medaka 3x	Indels	0.44
Nanopore	No	R10.3	sup	Canu	Indels	5.90
Nanopore	No	R10.3	sup	Flye_hq	Indels	3.01
Nanopore	No	R10.3	sup	Flye_hq+ medaka 1x	Indels	1.17
Nanopore	No	R10.3	sup	Flye_hq+ medaka 2x	Indels	1.13
Nanopore	No	R10.3	sup	Flye_hq+ medaka 3x	Indels	1.10
Nanopore	No	R10.4	hac	Canu	Indels	9.94
Nanopore	No	R10.4	hac	Flye_hq	Indels	4.57
Nanopore	No	R10.4	hac	Flye_hq+ medaka 1x	Indels	1.00
Nanopore	No	R10.4	hac	Flye_hq+ medaka 2x	Indels	0.98
Nanopore	No	R10.4	hac	Flye_hq+ medaka 3x	Indels	0.98
Nanopore	No	R10.4	sup	Canu	Indels	1.54
Nanopore	No	R10.4	sup	Flye_hq	Indels	0.99
Nanopore	No	R10.4	sup	Flye_hq+ medaka 1x	Indels	0.41
Nanopore	No	R10.4	sup	Flye_hq+ medaka 2x	Indels	0.41
Nanopore	No	R10.4	sup	Flye_hq+ medaka 3x	Indels	0.41
Nanopore	No	R10.4	duplex/sup	Canu	Indels	0.37
Nanopore	No	R10.4	duplex/sup	Flye_hq	Indels	0.48
Nanopore	No	R10.4	duplex/sup	Flye_hq+ medaka 1x	Indels	0.18
Nanopore	No	R10.4	duplex/sup	Flye_hq+	Indels	0.18

				medaka 2x		
Nanopore	No	R10.4	duplex/sup	Flye_hq+ medaka 3x	Indels	0.18

Figure S1. DNA extract tapestation profile. Neat DNA, measurements taken on 13/Sep/2021.

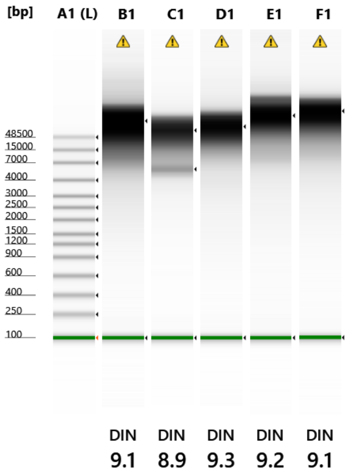

Figure S2. DNA extract tapestation profile. DNA diluted 10x, measurements taken on 13/Sep/2021.

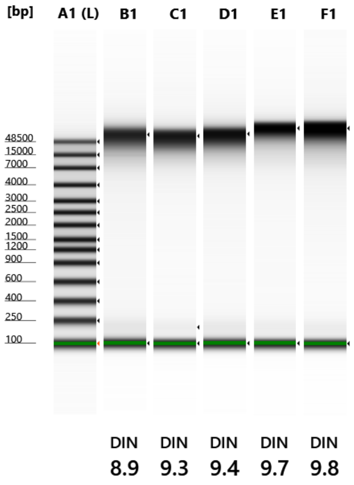

Figure S3. DNA extract tapestation profile. DNA diluted 10x, measurements taken on 31/Jan/2022.

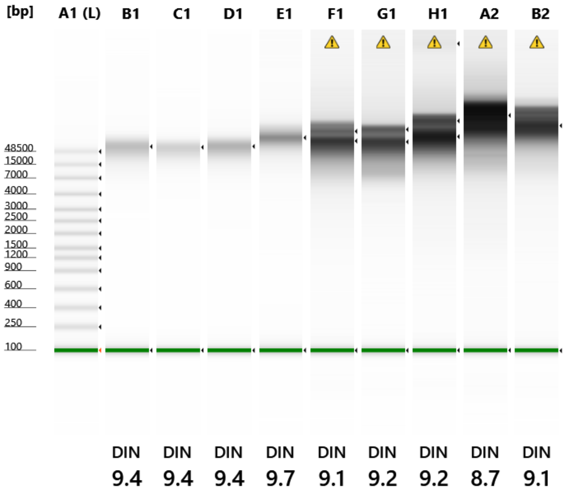

Figure S4. Sequencing yields over time for unplexed/multi(plexed) runs stratified by species and for R10.4, duplex versus simplex read outputs. Note y-axis scales (depicting yields in cumulative Gb) are different across facets.

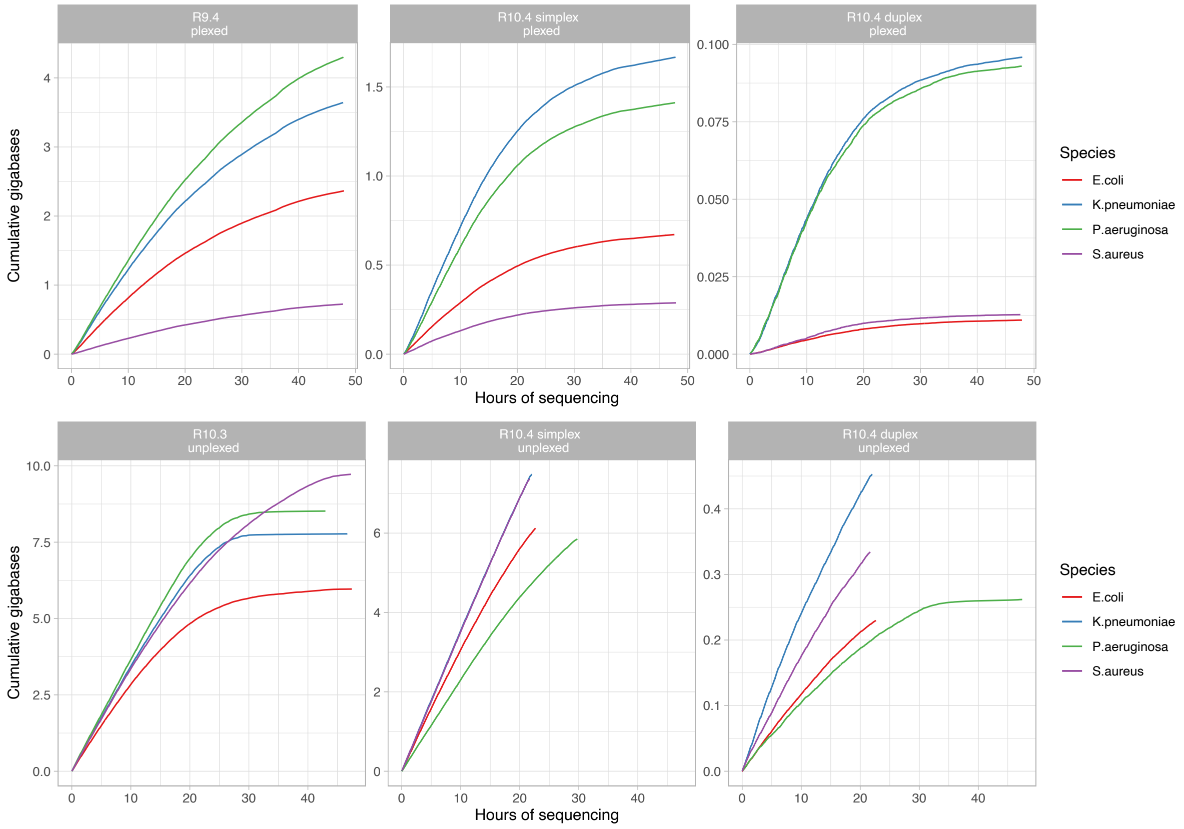

Figure S5. Impact of sub-sampling on number of contigs assembled for each species, modality and assembler - (A) chromosomes and (B) *K. pneumoniae* plasmids. In the plasmid plots, plasmids are denoted by their length in bp in the facet headers. For each comparison, there should be seven sub-sampling levels (represented by the rainbow colours) - if any of these are absent, then that contig was missing in the assembly. Similarly, in all plots, there should be single unique contig assembled (i.e. the y-axis should be 1). If this is >1, then the assembly is fragmented; or if 0, then the contig is absent.

A.

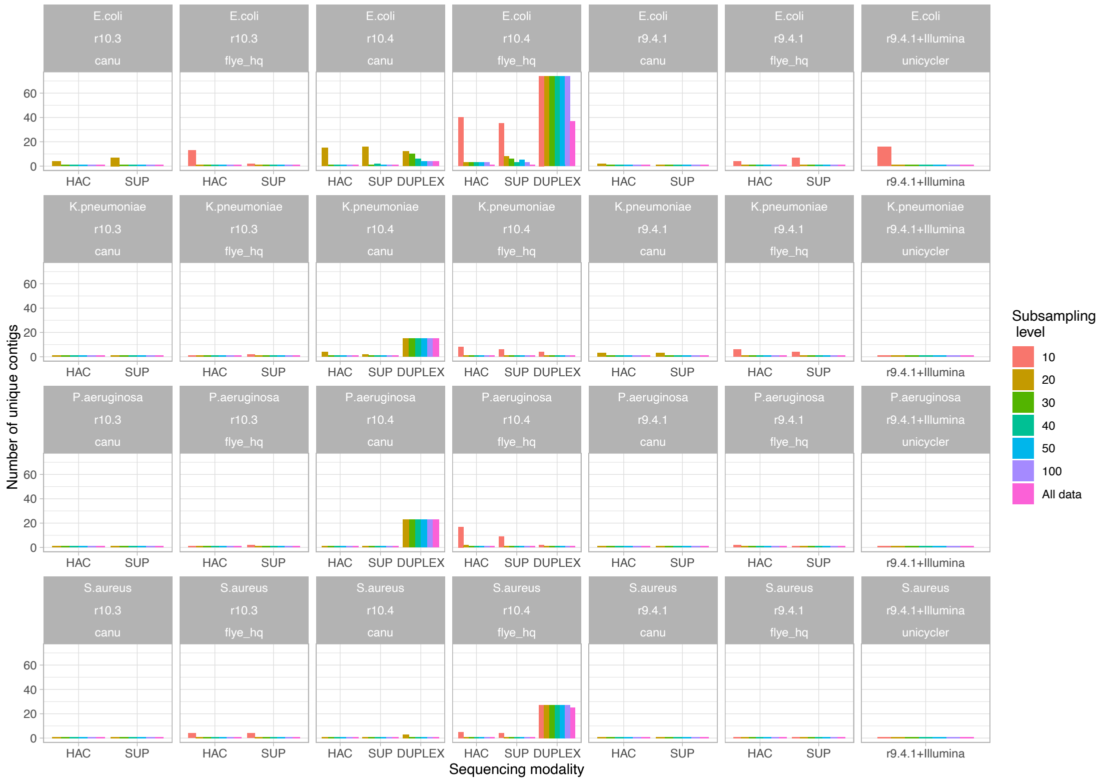

B.

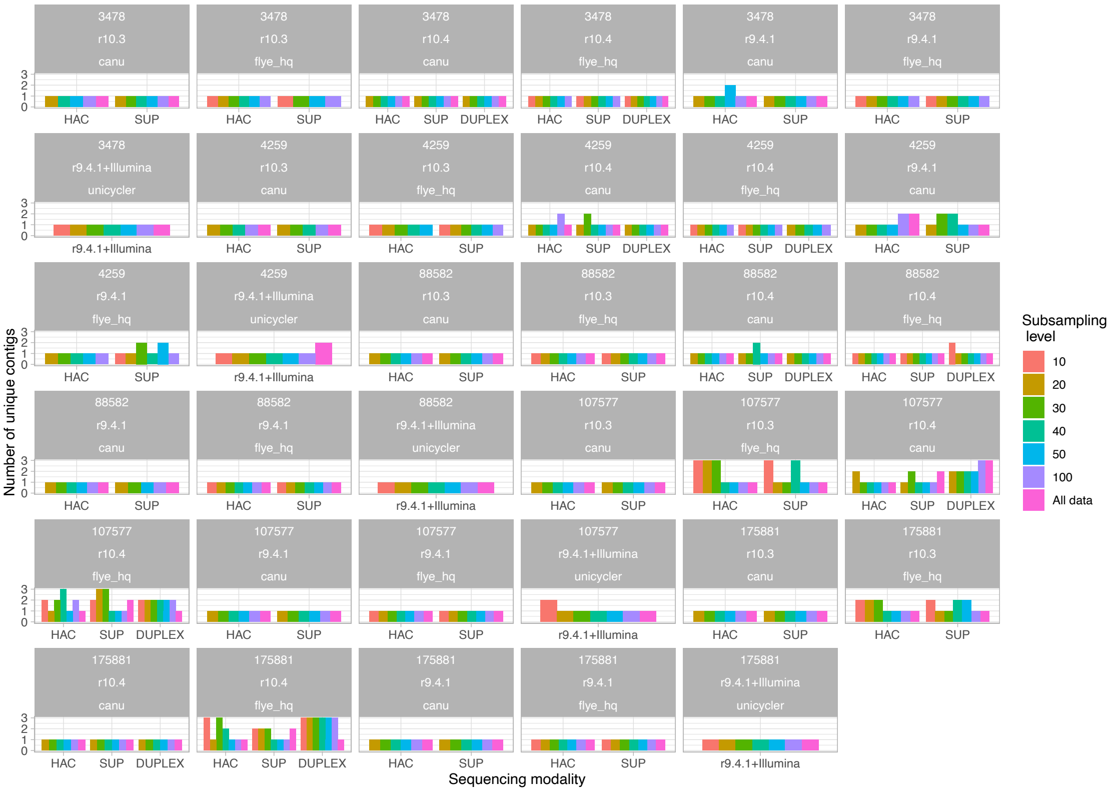

Figure S6. Impact of Medaka polishing of Flye assemblies, by species and read sub-sampling stratum.

A. *E. coli*

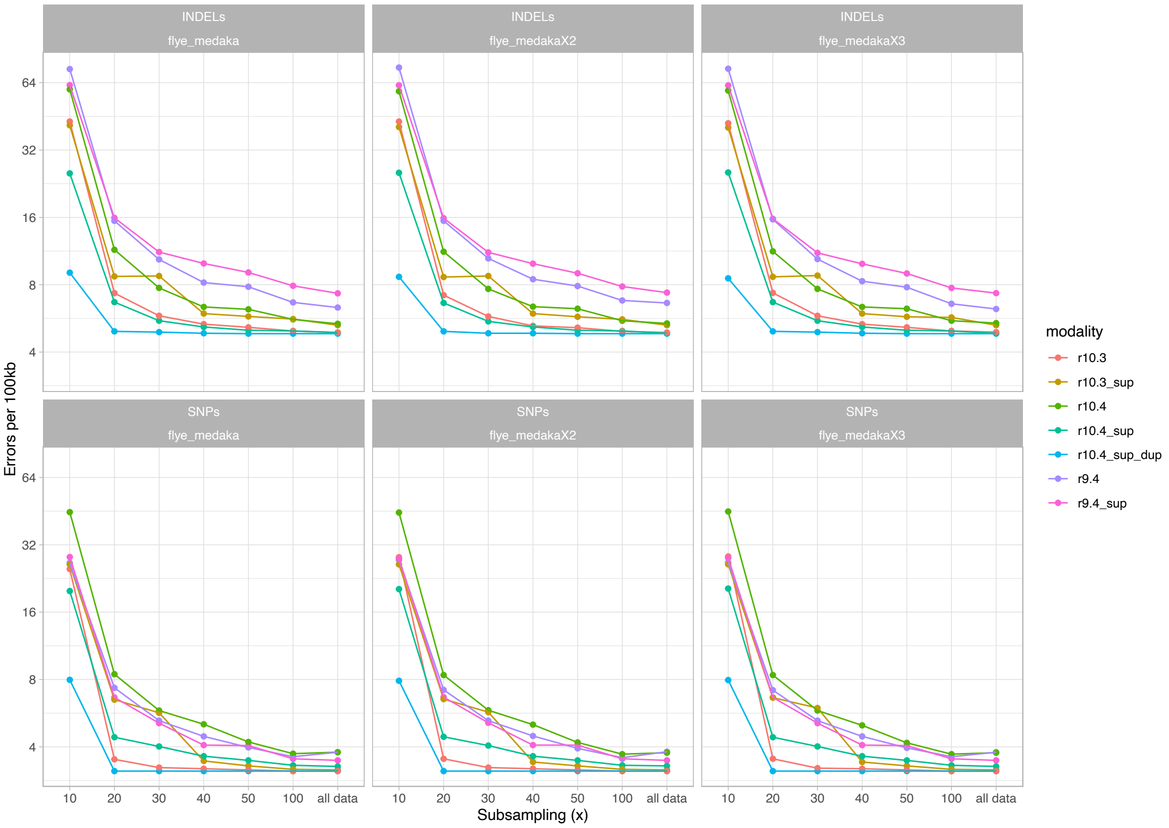

B. *K. pneumoniae*

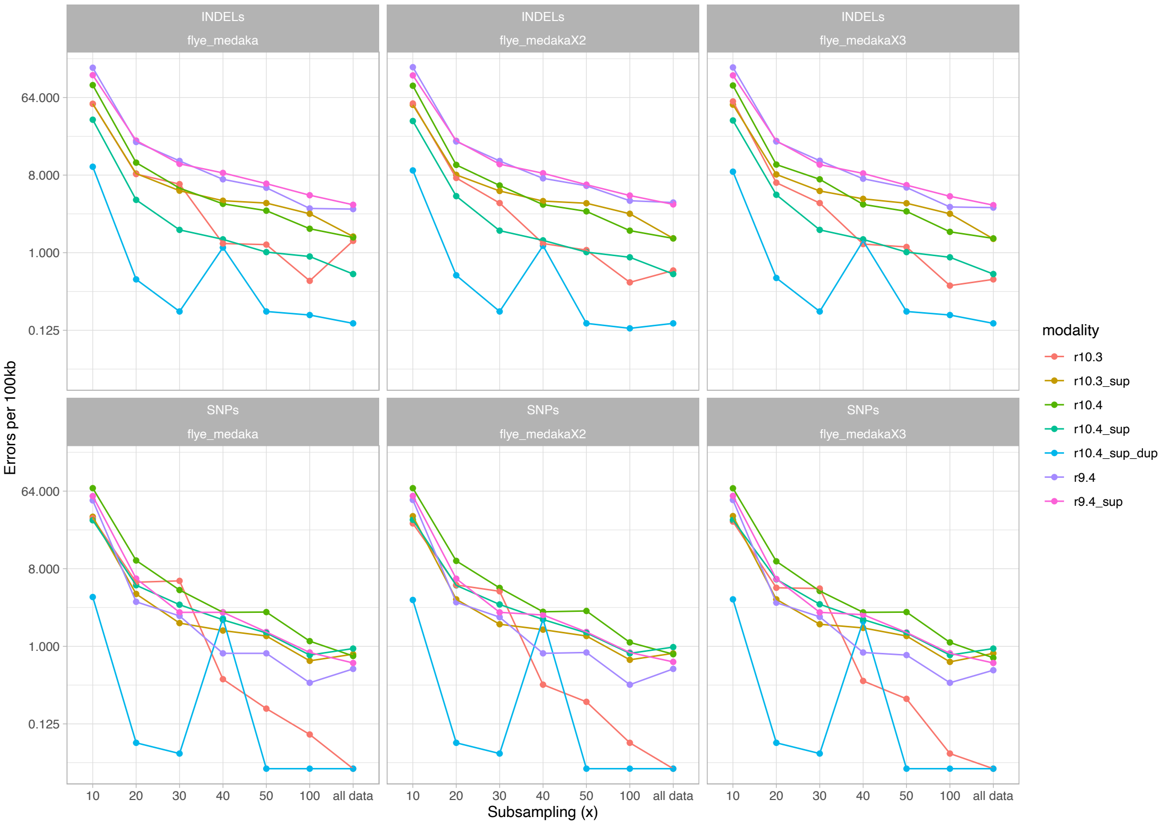

C. *P. aeruginosa*

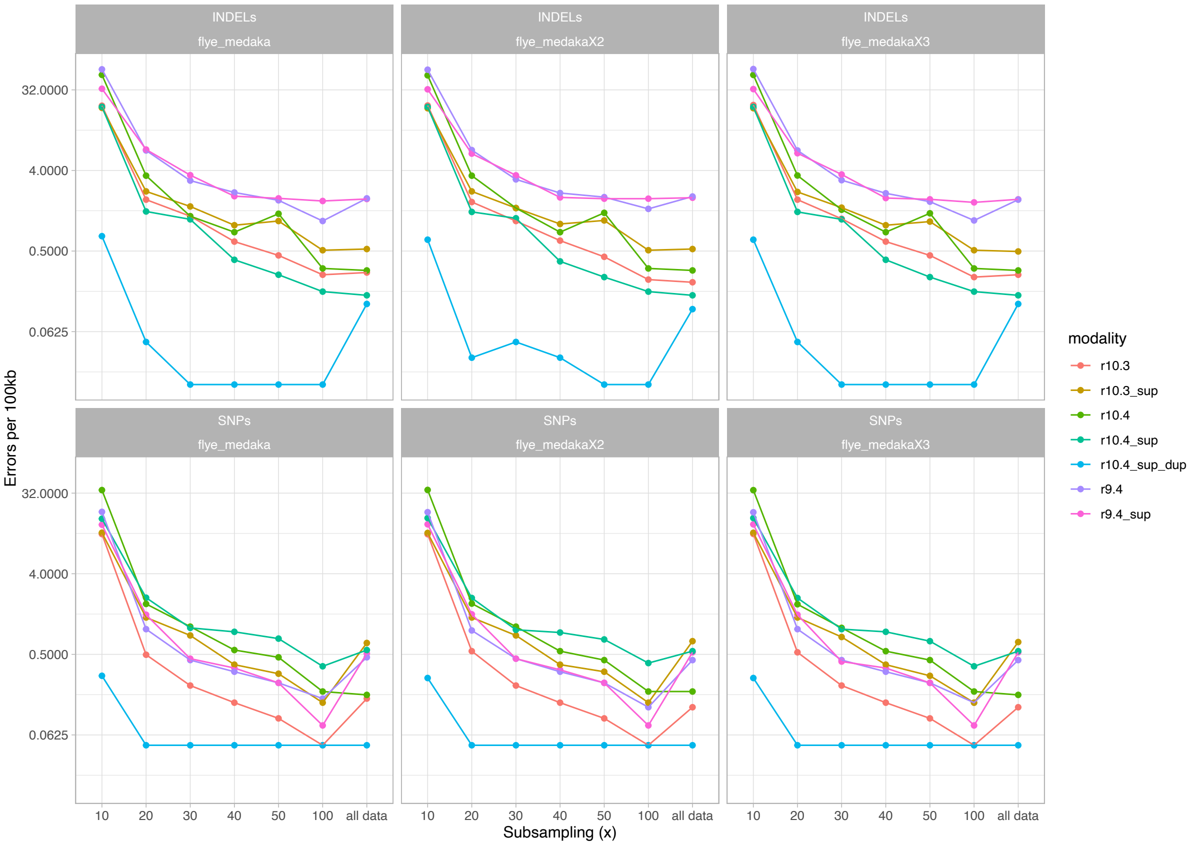

D. *S. aureus*

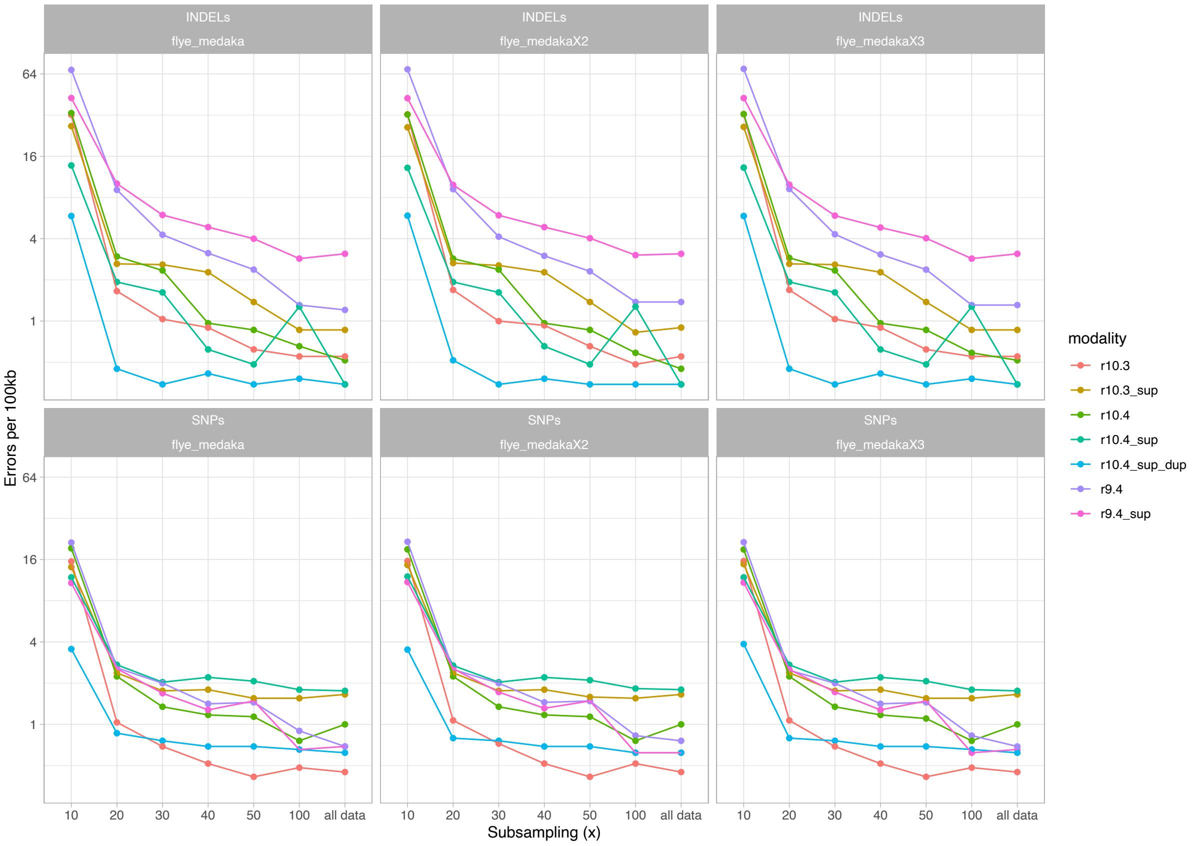

Supplement: Supplementary material 1 [file mgen-9-910-s001.pdf]
